# The complete chloroplast genome of tulip tree, *Liriodendron tulifipera* L. (Magnoliaceae): investigation of intra-species chloroplast variations

**DOI:** 10.1080/23802359.2019.1598822

**Published:** 2019-07-16

**Authors:** Jongsun Park, Yongsung Kim, Woochan Kwon, Hong Xi, Mi Kwon

**Affiliations:** a InfoBoss Co., Ltd., Gangnam-gu, Seoul, Korea;; b InfoBoss Research Center, Gangnam-gu, Seoul, Korea

**Keywords:** *Liriodendron tulifipera*, intra-species variations, chloroplast genome, Liriodendron, Magnoliaceae

## Abstract

*Liriodendron tulifipera* L. belongs to Magnoliaceae which is one of the basal angiosperm families. To understand intra-species variations on chloroplast genome in *Liriodendron* genus, we presented complete chloroplast genome of *L. tulifipera,* which is 156,387 bp long and has four subregions: 85,606 bp of large single copy (LSC) and 18,778 bp of small single copy (SSC) regions are separated by 26,002 bp of inverted repeat (IR) regions including 129 genes (84 coding genes, 8 rRNAs, and 37 tRNAs). The overall GC content of the chloroplast genome is 37.0% and those in the LSC, SSC, and IR regions are 34.9%, 30.5%, and 42.8%, respectively. Twelve single nucleotide polymorphisms (SNPs) located in one region and one insertion and deletion are found between *two L. tulifipera* genomes. INDEL Phylogenetic trees show that two *L. tulifipera* chloroplasts are clustered together and are sister to *Magnolia* species.

Genus *Liriodendron* is one of the genera belonging to Magnoliaceae, which is basal angiosperm family (Chase et al. 2016). Due to this importance, two *Liriodendron* chloroplast genomes were analyzed (Cai et al. 2006; Li et al. 2016). In addition, mitochondrial genomes of *Liriodendron tulifipera* have also been sequenced to show its phylogenetic position (Richardson et al. 2013; Park, Kim , Kwon, under review).

Intraspecies variations on chloroplast genome have been used for distinguishing its origins or cultivars (Selvaraj et al. 2008; Whittall et al. 2010; Huang et al. 2014; Ishizuka et al. 2017). Owing to the development of rapid development of sequencing technologies, organelle genomes, especially chloroplast, can be sequenced and assembled with simple processes and low costs. To understand intra-species variations on chloroplast of *L. tulifipera*, we completed second chloroplast genome of *L. tulifipera*.

Total DNA of *L. tulifipera* collected in the forest managed by Kookmin University in Korea was extracted from fresh leaves using DNeasy Plant Mini Kit (QIAGEN, Hilden, Germany). Genome sequencing was performed using HiSeq2000 at Macrogen Inc., Korea. Chloroplast genome was assembled and confirmed by Velvet 1.2.10 (Zerbino and Birney 2008), SOAPGapCloser 1.12 (Zhao et al. 2011), BWA 0.7.17 (Li 2013) and SAMtools 1.9 (Li et al. 2009). Geneious R11 11.0.5 (Biomatters Ltd, Auckland, New Zealand) was used for chloroplast genome annotation based on *L. tulifipera* chloroplast genome (NC_008326).

The chloroplast genome of Korean *L. tulifipera* (Genbank accession is MK477550) is 159,885 bp long (GC ratio is 39.2%) and has four subregions: 85,606 bp of large single copy (LSC; 34.9%) and 18,778 bp of small single copy (SSC; 30.5%) regions are separated by 26,002 bp of inverted repeat (IR; 42.8%), which is longer than that of *L.* tulifipera by 1 bp (Cai et al. 2006). It contains 129 genes (84 protein-coding genes, 8 rRNAs, and 37 tRNAs); 18 genes (7 protein-coding genes, 4 rRNAs, and 7 tRNAs) are duplicated in IR regions.

Based on pair-wise alignment of both *Liriodendron* chloroplasts, 12 single nucleotide polymorphisms (SNPs) and one insertion and deletion (INDEL) are identified. This amount of intra-species sequence variations is larger than those of *Coffea* (Park, Xi et al., under review; Park, Kim, Xi, Nho et al., under review) and *Marchantia* (Kwon et al. under review), similar to that of *Nymphaea* (Park, Kim, Kwon et al. under review) and smaller than those of *Duchesnea* (Park, Kim, Lee, under review), *Pseudostellaria* (Kim et al., under review), *Camellia* (Park, Kim, Xi, et al. under review), *Rehmannia* (Jeon et al. 2019), and *Illicium* (Park, Kim, Xi, under review) . Interestingly, all 12 SNPs are found continuously at 67,837 bp to 67,848 bp, which is different from normal SNPs. One INDEL is located in IR-SSC boundary.

Fourteen Magnoliaceae containing two *L. tulifipera* chloroplasts, four Nymphaceae, and one *Amborella* chloroplast genomes as an outgroup were used for drawing neighbor joining (bootstrap repeat is 10,000) and maximum likelihood (bootstrap repeat is 1000) trees using MAFFT 7.388 (Katoh and Standley 2013) and MEGA X (Kumar et al. 2018). Phylogenetic trees show that two *L. tulifipera* chloroplasts are clustered together and are sister to *Magnolia* species (Figure 1).

**Figure 1.**
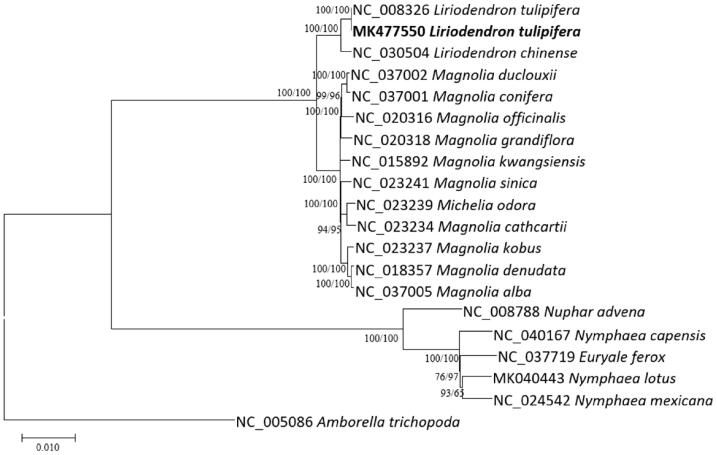
Neighbor joining (bootstrap repeat is 10,000) and maximum likelihood (bootstrap repeat is 1,000) phylogenetic trees of 14 Magnoliaceae, 4 Nymphaceae, and 1 *Amborella* complete chloroplast genomes: two *Liriodendron tulifipera* (MK477550 in this study and NC_008326), *Liriodendron chinense* (NC_030504), *Magnolia alba* (NC_037005), *Magnolia cathcartii* (NC_023234), *Magnolia conifera* (NC_037001), *Magnolia denudata* (NC_018357), *Magnolia duclouxii* (NC_37002), *Magnolia grandiflora* (NC_020318), *Magnolia kobus* (NC_023237), *Magnolia kwangsiensis* (NC_015892), *Magnolia officinalis* (NC_020316), *Magnolia sinica* (NC_023241), *Michelia odora* (NC_023239), *Euryale ferox* (NC_037719), *Nuphar advena* (NC_008788), *Nymphaea capensis* (NC_040167), *Nymphaea lotus* (MK040443), *Nymphaea mexicana* (NC_024542), and *Amborella trichopoda* (NC_005086). The numbers above branches indicate bootstrap support values of maximum likelihood and neighbor joining phylogenetic trees, respectively.
